# “Iron-saturated” bovine lactoferrin improves the chemotherapeutic effects of tamoxifen in the treatment of basal-like breast cancer in mice

**DOI:** 10.1186/1471-2407-12-591

**Published:** 2012-12-11

**Authors:** Xueying Sun, Ruohan Jiang, Aneta Przepiorski, Shiva Reddy, Kate P Palmano, Geoffrey W Krissansen

**Affiliations:** 1 Department of Molecular Medicine & Pathology, Faculty of Medical and Health Sciences, University of Auckland, Auckland, 1005, New Zealand; 2 Fonterra Research Centre, Palmerston North, 4442, New Zealand; 3 Department of General Surgery, The Hepatosplenic Surgery Center, The First Affiliated Hospital of Harbin Medical University, Harbin, 150001, China

**Keywords:** Breast cancer, Iron-saturated lactoferrin, Tamoxifen, Immune enhancement, Mice

## Abstract

**Background:**

Tamoxifen is used in hormone therapy for estrogen-receptor (ER)-positive breast cancer, but also has chemopreventative effects against ER-negative breast cancers. This study sought to investigate whether oral iron-saturated bovine lactoferrin (Fe-Lf), a natural product which enhances chemotherapy, could improve the chemotherapeutic effects of tamoxifen in the treatment of ER-negative breast cancers.

**Methods:**

In a model of breast cancer prevention, female Balb/c mice treated with tamoxifen (5 mg/Kg) were fed an Fe-Lf supplemented diet (5 g/Kg diet) or the base diet. At week 2, 4T1 mammary carcinoma cells were injected into an inguinal mammary fat pad. In a model of breast cancer treatment, tamoxifen treatment was not started until two weeks following tumor cell injection. Tumor growth, metastasis, body weight, and levels of interleukin 18 (IL-18) and interferon γ (IFN-γ) were analyzed.

**Results:**

Tamoxifen weakly (IC_50_ ~ 8 μM) inhibited the proliferation of 4T1 cells at pharmacological concentrations *in vitro*. In the tumor prevention study, a Fe-Lf diet in combination with tamoxifen caused a 4 day delay in tumor formation, and significantly inhibited tumor growth and metastasis to the liver and lung by 48, 58, and 66% (all *P* < 0.001), respectively, compared to untreated controls. The combination therapy was significantly (all *P* < 0.05) more effective than the respective monotherapies. Oral Fe-Lf attenuated the loss of body weight caused by tamoxifen and cancer cachexia. It prevented tamoxifen-induced reductions in serum levels of IL-18 and IFN-γ, and intestinal cells expressing IL-18 and IFN-γ. It increased the levels of Lf in leukocytes residing in gut-associated lymphoid tissues. B, T and Natural killer (NK) cells containing high levels of Lf were identified in 4T1 tumors, suggesting they had migrated from the intestine. Similar effects of Fe-Lf and tamoxifen on tumor cell viability were seen in the treatment of established tumors.

**Conclusions:**

The results indicate that Fe-Lf is a potent natural adjuvant capable of augmenting the chemotherapeutic activity of tamoxifen. It could have application in delaying relapse in tamoxifen-treated breast cancer patients who are at risk of developing ER-negative tumors.

## Background

Breast cancer is the most common cause of cancer death in women worldwide [1]. Tamoxifen has been employed for over 20 years as the drug of choice for the treatment of estrogen receptor positive (ER^+ve^) breast cancer [2,3]. Despite providing a considerable initial benefit to at least half of all patients, the majority of breast cancers eventually become resistant to the cytostatic effects of tamoxifen within 5 years of treatment [4], leading to an increased risk of development of ER^-ve^ breast cancers [4-6], particularly contralateral cancers [7]. The outgrowth of triple negative “basal-like” tumor cells lacking the ER, progesterone receptor (PR), and human epidermal growth factor receptor 2 (HER2) is particularly concerning as patients with these tumors have a poor prognosis [8]. Loss of effectiveness of tamoxifen is problematic for breast cancer survivors undergoing long-term therapy as tamoxifen inhibits the immune response which might otherwise help to keep their cancers in-check. Tamoxifen treatment downregulates the expression of the cytokine interleukin (IL)-18 [9], lowers the numbers of CD4^+^ T cells [10], and reduces natural killer (NK) cell activity [10]. It inhibits the functions of monocytes, antibody formation, dendritic cell differentiation and activation, and reduces lymphoid organ weights in rodents [11-14]. It upregulates the expression of the potently immunosuppressive cytokine transforming growth factor (TGF)-β1 in breast tumors, which tumors use to avoid the immune response, and is implicated in the failure of tamoxifen therapy [15]. Upregulation of TGF-β1 is also seen with the ER antagonist fulvestrant, suggesting it may be a common feature of several anti-estrogens [16].

The potential detrimental effects that tamoxifen has on patients at risk of developing ER^-ve^ breast cancers might be worse were it not for the fact that tamoxifen displays chemopreventative activity, due to off target effects. Like many small molecule inhibitors, tamoxifen is not a highly selective drug. It has been reported to display anti-tumor activity against ER^-ve^ breast cancers, and other unrelated cancers [17-19]. Pharmacological concentrations of tamoxifen induce proapoptotic effects in ER^-ve^ breast cancer cells, via the activation/inactivation of signaling pathways that involve phosphatidylinositol 3-kinase (PI3K)/Akt, extracellular-signal-regulated kinase (ERK), and insulin-like growth factor 1 receptor (IGF-1R) [20]. The chemopreventative effects of tamoxifen against ER^-ve^ breast cancer cells and tumors have been demonstrated by using tamoxifen alone or in synergistic combinations with various natural products and chemical agents including epigallocatechin gallate [21], docetaxel, genistein, black cohosh, palm oil tocotrienols, OSU-03012 (latter studies are cited in ref 21), roscovitine [22], persin [23], flax seed enterodiol and enterolactone [24], mifepristone [25], interferons [26] and tumor necrosis factor-related apoptosis-inducing ligand (TRAIL) [27]. Tamoxifen in combination with paclitaxel has a cytotoxic effect against ER^-ve^ colon cancer and lung cancer cell lines [28]. One approach to bolster the chemopreventative effects of tamoxifen is to use immunotherapy, which may help overcome tamoxifen-induced immunosuppression. Thus, IFN-γ and IL-2 immunotherapy significantly improved the clinical response and survival of breast cancer patients treated with tamoxifen [29,30].

Lactoferrin (Lf) is an iron-binding glycoprotein present in bodily secretions, which serves as a natural antibiotic, but also has anti-tumor activity [31,32]. Lf-induced anti-tumor activity was lost in mice depleted of CD8^+^ T cells and in CD1 knockout mice lacking NK T cell activity, suggesting Lf functions by stimulating anti-tumor immunity [33]. Oral Lf accelerated reconstitution of humoral and cellular immune responses during chemotherapy-induced immunosuppression in mice [34,35], suggesting it could be employed to overcome tamoxifen-induced immune suppression.

We recently showed that iron-saturated Fe-Lf was superior to natural bovine Lf (bLf) in stimulating anti-tumor immunity and inhibiting tumor growth, especially when used in combination with chemotherapy [36]. Further, it reduced the side-effects of chemotherapy by restoring red and white blood cell counts. Here we investigated the ability of Fe-Lf to improve the chemotherapeutic effects of tamoxifen against 4T1 tumors that express low levels of ER, PR, and HER2, and represent a mouse model of intractable, basal-like, metastatic breast cancer.

## Methods

### Mice and cells

Female 6–8 week old Balb/c mice were obtained from the Animal Resource Unit, Faculty of Medical and Health Sciences, University of Auckland, Auckland, New Zealand. They were kept in an air-conditioned room with controlled humidity, temperature, and 12 h light: dark cycle. All experiments were conducted under a protocol approved by the Animal Ethics Committee, University of Auckland. The mouse 4T1 mammary carcinoma cell line (Balb/c origin), which was purchased from the American Type Culture Collection (Rockville, MD, USA) very weakly expresses the ER [37] and is non-responsive to estrogen [38]. Tamoxifen at 5 μg/ml significantly inhibited the viability of 4T1 cells in culture at 48, 72, 96, and 120 hour time periods, and significantly increased the life-span of mice inoculated with 4T1 tumor cells [39].

### Antibodies

The primary Abs used in this study included a mouse anti-bovine Lf Ab (Hycult Biotechnology, Frontstraat 2a, 5405 PB Uden, The Netherlands), a rat anti-mouse CD11b Ab (monocyte/macrophage marker, BD Biosciences, NJ), a mouse anti-mouse PK136 Ab (NK cell marker, Biolegend, San Diego, CA), rat anti-mouse IL-18 and IFN-γ Abs (BD Biosciences), a rat anti-mouse CD3 Ab (T cell marker, Biolegend), a rat anti-mouse B cell marker Ab (Serotec, Oxford, UK), and a rat anti-mouse dendritic cell marker Ab (eBioscience, San Diego, CA). The secondary Abs used in this study included a fluorescein isothiocyanate (FITC)-conjugated rat anti-mouse IgG (Sigma), an alexa fluor 568-conjugated donkey anti-mouse Ab and an alexa fluor 568-conjugated goat anti-rat Ab (Invitrogen, Auckland, New Zealand).

### Experimental diets

Bovine Lf that had been saturated with iron to 100% using an industrial scale food grade method was provided by Fonterra Co-operative Group Limited, New Zealand. The experimental diets were prepared according to the Harlan Teklad AIN93M base formulation. The Fe-Lf diet was produced by partial substitution of the casein component of the control diet with Fe-Lf (5 g/Kg diet), such that the total protein content of the diet was unchanged. The compositions of the control and Fe-Lf diets are shown in Table 1. The mice were provided with fresh diet thrice per week, and they had free access to food and water throughout the study.

**Table 1:** Compositions of experimental diets*

**Component (g/Kg)**	**Control diet**	**Fe-Lf diet**
Casein	144.3	139.3
Fe-Lf	0	5
L-Cystine	1.79	1.79
Corn starch	463.38	463.38
Maltodextrin	154.23	154.23
Sucrose	99.5	99.5
Soybean oil	39.8	39.8
Cellulose	49.75	49.75
Mineral mix, AIN-93 M-MX (94049)	34.83	34.83
Vitamin mix, AIN-93-VX (94047)	9.95	9.95
Choline bitartrate	2.49	2.49
TBHQ, antioxidant	0.008	0.008

*The diets were prepared based on the Harlan Teklad AIN-93 M

(TD 94048) diet. TBHQ, tert-butylhydroquinone; Fe-Lf, iron-saturated bovine lactoferrin (100% iron-saturated).

### Experimental animal models and treatments

**Figure 1:**
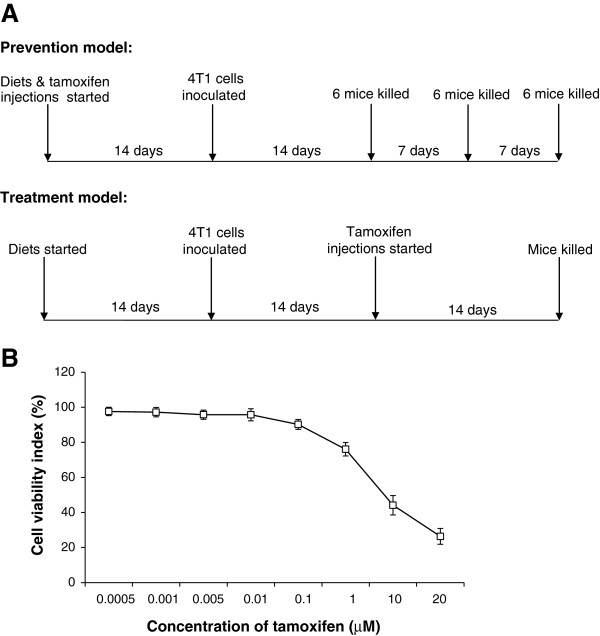
**Experimental protocols for prevention and treatment models of breast cancer, and sensitivity of 4T1 cells to tamoxifen. A**: Experimental protocols. In the prevention model, mice were placed on the control diet or the Fe-Lf diet, and 4T1 tumor cells were injected into the mammary fat pad 14 days later. Tamoxifen or PBS was administered i.p. on the day the mice were placed on their diets, and on alternate days thereafter. Six mice per group (n = 18) were randomly killed at the indicated time points. In the treatment model, the mice were placed on the diets and 4T1 cells were injected into the mammary fat pad 14 days later. Tamoxifen or PBS was administered 14 days after injection of tumor cells, and on alternate days thereafter. Each group had 6 mice, which were killed at the completion of the experiment. **B**: Tamoxifen has chemotherapeutic effects against 4T1 cells. 4T1 cells were incubated with increasing concentrations of tamoxifen, and their viability assessed 72 h later by the MTT assay. The cell viability index (% viability) was plotted versus the concentration of tamoxifen.

In the prevention experiment, 72 six-week-old Balb/c female mice were randomized into four groups of 18 animals each, to receive either the control diet, control diet + tamoxifen, Fe-Lf diet, or Fe-Lf diet + tamoxifen. The feeding schedules are shown in Figure 1A. In the control diet and Fe-Lf diet groups, the mice were fed with control or Fe-Lf diets, respectively, and received an i.p. injection of 100 μL of PBS every two days. In the control diet + tamoxifen and Fe-Lf diet + tamoxifen groups, the mice were fed with control and Fe-Lf diets, respectively, and received an injection of 100 μL of tamoxifen (Sigma, MO) suspension at a dose of 5 mg/Kg body weight every two days. The tamoxifen powder was initially dissolved in 100% ethanol, and then diluted in PBS to prepare a tamoxifen injectable suspension. Tamoxifen was injected subcutaneously on the inside of either thigh with the sites of injection being rotated. Fourteen days later, 50 μl of a mixture of BD Matrigel™ Basement Membrane Matrix (BD Biosciences) and PBS (phosphate buffered saline) (1:1, v/v) containing 2 × 10^4^ 4T1 cells was injected into the right inguinal mammary fat pad of mice. The mice were monitored and weighed, and the sizes of the tumors were recorded by measuring tumor diameters. Six mice in each group were killed at the indicated time points (Figure 1A), bled by cardiac puncture and sera isolated. Tumors, lungs, livers, small intestines, gastrocnemius muscles and ovarian adipose tissues were excised and weighed.

In the treatment experiment (Figure 1A), 24 mice were randomized into four groups of 6 mice as in the prevention experiment, but the injections of tamoxifen or PBS were started when the tumors reached ~0.2 to 0.3 cm in diameter, 14 days after injection of 4T1 cells.

### Measurement of tumor metastases

The numbers of metastatic tumors on the lung surface were counted. The livers were fixed with 4% buffered formalin solution and transverse 5-μm sections were prepared at 5 different levels to cover the entire liver. The sections were stained with haematoxylin and eosin (HE), metastatic nodules containing more than 6 cancer cells were counted, and the mean number of nodules was recorded as the number of metastases.

### Immunohistochemical analysis

Formalin-fixed tissues were embedded in paraffin and sectioned. After antigen retrieval, the slides were rehydrated, and blocked with 5% casein in PBS containing 2% normal horse serum or 2% BSA (bovine serum albumin) at 4°C overnight. The sections were incubated with primary Abs overnight at 4°C, followed by incubation with appropriate secondary Abs for 1 h at room temperature. They were then washed and mounted, and examined using a Nikon E600 fluorescent microscope.

### Enzyme-linked immunosorbent assay (ELISA)

Serum levels of IL-18 and IFN-γ were measured with mouse IL-18 and IFN-γ ELISA kits (R&D Systems), respectively.

### MTT assay

4T1 cells (2 × 10^3^) were seeded in 200 μl of RPMI 1640 medium into 96-well plates, and cultured overnight. The medium was replaced with the fresh RPMI 1640 medium or the same media containing tamoxifen. After a further incubation for 72 h, methyl thiazolyl tetrazolium (MTT) (20 μl) was added to each well followed by a 4 h incubation. The medium was discarded and 150 μl of dimethyl sulfoxide (DMSO) was added into each well, and incubated for 20 min. The optical density (OD) was measured at 490 nm. The cell viability index was calculated according to the formula: experimental OD value/control OD value × 100%. The experiments were repeated thrice.

### Statistical analysis

Results were expressed as mean values ± standard deviation (SD). A one way analysis of variance (ANOVA) followed by Dunnett’s test (PASW statistics 18) was used for evaluating statistical significance. *P* < 0.05 was considered to be statistically significant.

## Results

### Tamoxifen reduces the viability of 4T1 cells *in vitro*

The chemotherapeutic effect of tamoxifen on the viability of 4T1 cells was examined by incubating 4T1 cells with different concentrations of tamoxifen for 72 h. As shown in Figure 1B, tamoxifen at concentrations of 1 μM and greater inhibited the viability of 4T1 cells, with an IC_50_ of 8.1 μM.

### Bovine Fe-Lf augments tamoxifen therapy to inhibit the formation and growth of basal-like breast tumors

**Figure 2:**
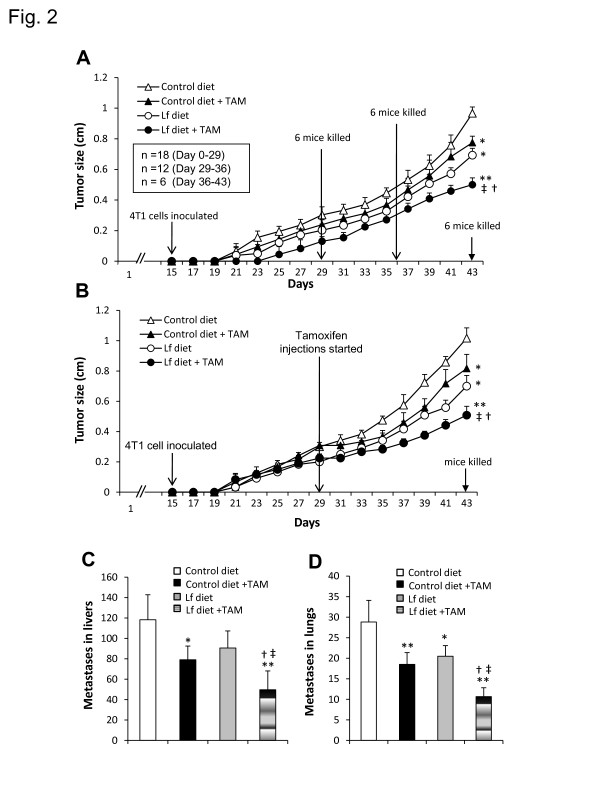
**Fe-Lf augments tamoxifen therapy to suppress the formation and growth of 4T1 tumors and their metastasis to livers and lungs. A**,**B**: Fe-Lf augments tamoxifen therapy to suppress the formation and growth of 4T1 tumors. **A**: In the prevention experiment, 6 mice from each group were randomly killed for sampling on days 29, 36, and 43 following placement on diets and the start of administration of tamoxifen (TAM). Tumor size was measured every two days. **B**: In the treatment experiment, tamoxifen (TAM) was administered to the mice 14 days after injection of tumor cells. Each group had 6 mice, and tumor size was measured every two days. **C,D**: Fe-Lf augments tamoxifen therapy to suppress metastasis to livers and lungs. Mice in the prevention experiment were euthanized on day 43, and their livers and lungs removed. The livers were sectioned and stained with HE. The numbers of metastatic tumor nodules in liver sections (**C**) and the number of metastatic tumors on the surface of lungs (**D**) were counted, respectively. Results are expressed as the mean value ± SD. “*” *P* < 0.05 or “**” *P* <0.001 versus the group fed the control diet, “†” *P* < 0.05 versus the group fed the control diet and treated with tamoxifen, and “‡” *P* < 0.05 versus the group fed the Fe-Lf diet. **E**: Fe-Lf attenuates loss of body weight caused by cancer cachexia and tamoxifen therapy, and inhibits tamoxifen-induced reductions of IL-18 and IFN-γ in sera and intestinal cells. The mice in the prevention experiment were weighed every two days. “*” *P* < 0.05 versus the group fed the control diet, “†” *P* < 0.05 versus the group fed the control diet and treated with tamoxifen (TAM).

Groups of Balb/c mice were placed on either the control diet or the Fe-Lf diet, and received injections of either PBS or tamoxifen every two days to determine whether Fe-Lf would augment the effects of tamoxifen in preventing the formation of breast tumors (Figure 1A). Fourteen days after starting the treatments, 4T1 breast tumor cells were injected into the right inguinal mammary fat pad. Neither the Fe-Lf nor tamoxifen monotherapies delayed the appearance of palpable 4T1 tumors, whereas in contrast the combination of the Fe-Lf diet and tamoxifen delayed the appearance of palpable tumors by 4 days, and inhibited their growth compared with the control diet (Figure 2A). Consequently, on day 43 the tumors formed were on average 48% smaller (*P* < 0.001) than the tumors in the control diet group, and were significantly (*P* < 0.05) smaller than tumors of the monotherapy groups (Figure 2A). Nevertheless, each of the Fe-Lf and tamoxifen monotherapies inhibited tumor growth, resulting in significantly (both *P* < 0.05) smaller tumors on day 43 than the tumors of mice fed the control diet. The size of tumors was in accordance with the weight of tumors as shown in Table 2. To investigate whether the effects of the combination of the Fe-Lf diet and tamoxifen were synergistic, we calculated the value for the coefficient of drug interaction (CDI), as described previously [40]. The CDI value on day 43 was 0.9 (less than 1), indicating that Fe-Lf and tamoxifen have a synergistic effect in inhibiting tumor growth.

**Table 2:** **Body, tumor, organ and tissue weights**^**1**^

	**Control diet**	**Control diet + TAM**	**Lf diet**	**Lf diet + TAM**
	**(n = 6)**	**(n = 6)**	**(n = 6)**	**(n = 6)**
Body weight (g)	16.9 ± 1.1	13.9 ± 0.4^2^	18.1 ± 1.1	15.0 ± 0.5^2,3,4^
Carcass body weight (g)^5^	16.4 ± 1.3	13.6 ± 0.4^2^	18.0 ± 1.2^2^	14.8 ± 0.5^2,3,4^
Tumor (mg)^6^	309.4 ± 32.9	216.3 ± 26.7^2^	194.7 ± 19.8^2^	146.2 ± 17.3^2,3,4^
Liver (mg)^7^	1262.5 ± 44.1	1215.7 ± 109.5	1318.2 ± 40.8	1285.2 ± 86.9
Lung (mg)^8^	389.2 ± 37.1	297.2 ± 19.4^2^	309.3 ± 27.9^2^	281.3 ± 22.2^2^
Gastrocnemius muscle (mg)	91.8 ± 6.7	77.9 ± 8.2	99.6 ± 4.2	88.4 ± 5.0^2,3,4^
Ovarian adipose tissue (mg)	26.1 ± 2.5	22.5 ± 1.7^2^	28.1 ± 1.3	25.0 ± 1.8^3^

^1^Tumors, lungs, livers, small intestines, gastrocnemius muscles and ovarian adipose tissues of mice in the prevention study were excised at day 43 and weighed. Data are expressed as means ± SD. Statistical significance was determined by one way ANOVA followed by Dunnett’s test. ^2^*P* < 0.05 versus the mice fed the control diet; ^3^*P* < 0.05 versus the mice fed the control diet and treated with TAM; ^4^*P* < 0.05 versus the mice fed Lf diet. ^5^Calculated according to the formula: body weight – tumor weight. ^6^Tumor refers to primary tumor. ^7^Liver weight includes metastatic tumors. ^8^Lung weight includes metastatic tumors. TAM, tamoxifen.

In the treatment experiment (Figure 2B), mice were placed on their diets, and 14 days later 4T1 cells were injected into a mammary fat pad. They received injections of either PBS or tamoxifen every two days when their tumors reached ~0.2 to 0.3 cm in diameter 14 days after injection of the 4T1 cells. Similar results were obtained as in the prevention experiment, where the Fe-Lf and tamoxifen therapies each significantly (*P* < 0.05) suppressed the growth of tumors (Figure 2B). Again the combination of Fe-Lf and tamoxifen proved to be the most effective, having a significant (*P* < 0.05) effect compared to the monotherapies, with a CDI of 0.9.

### Fe-Lf augments tamoxifen therapy to suppress the dissemination of tumor metastases to the liver and lung

#### Suppression of liver metastases

The 4T1 breast cancer cell line is highly metastatic and disseminates to the lung and liver while the primary tumor is growing *in situ*[41]. The livers of mice in the prevention experiment (Figure 2A; day 43) were sectioned and stained, and the numbers of metastatic nodules inside the livers were counted. The mean number of metastases in the liver sections of untreated mice fed the control diet, tamoxifen-treated mice fed the control diet, untreated mice fed the Fe-Lf diet, and tamoxifen-treated mice fed the Fe-Lf diet, was 118, 76, 91 and 50, respectively (Figure 2C). Thus, tamoxifen therapy and the Fe-Lf diet each significantly (*P* < 0.05) reduced the numbers of tumors in the liver by 36% and 23%, respectively, compared with untreated mice fed the control diet. The Fe-Lf diet in combination with tamoxifen therapy was the most effective, reducing tumor numbers by 58% (*P* < 0.001), 45% (*P* < 0.05), and 34% (*P* < 0.05), compared with untreated mice fed the control diet, untreated mice fed the Fe-Lf diet, and tamoxifen-treated mice fed the control diet, respectively.

#### Suppression of lung metastases

The surfaces of the lungs of the mice in the prevention experiment (Figure 2A; day 43) were inspected for the presence of metastatic 4T1 tumors. The mean number of metastatic tumors on the lungs of untreated mice fed the control diet, tamoxifen-treated mice fed the control diet, untreated mice fed Fe-Lf diet, and tamoxifen-treated mice fed Fe-Lf diet, was 29, 19, 21 and 11, respectively (Figure 2D). Thus, tamoxifen treatment and the Fe-Lf diet each significantly (*P* < 0.01) reduced the numbers of tumors on the lung surface by 34% and 28%, respectively, compared with untreated mice fed the control diet. The Fe-Lf diet in combination with tamoxifen therapy was the most effective, reducing tumor numbers by 66% (P < 0.001), 48% (P < 0.05) and 42% (P < 0.05), respectively, compared with untreated mice fed the control diet, untreated mice fed the Fe-Lf diet, and tamoxifen-treated mice fed the control diet. The numbers of lung metastases were in accordance with the weight of the lungs, where increased numbers of metastases correlated with increased organ weight, as shown in Table 2.

### Oral Fe-Lf attenuates loss of body weight caused by cancer cachexia and tamoxifen therapy

The 4T1 tumor model represents a model of late-stage breast cancer and cancer cachexia. The body weights of all four groups of mice in the prevention experiment (Figure 2A) began to decline once the tumors reached around 0.2 cm in diameter at day 29, possibly because of the increasing cachectic status of the mice (Figure 2E). Untreated tumor-bearing mice fed the control diet experienced a significant (*P* < 0.05) 12% reduction in carcass body weight at day 43 compared with day 29 (Figure 2E), as reflected by significant losses in the weights of gastrocnemius muscle and ovarian adipose tissues (Table 2). Feeding of the Fe-Lf diet attenuated the cachectic status of mice. Thus, mice fed the Fe-Lf diet had significantly (*P* < 0.05) higher body weights compared to the mice fed the control diet (Figure 2E), as reflected by significantly higher carcass weights (Table 2).

Tamoxifen has an effect on energy homeostasis in rodents such that it markedly decreases food intake and body weight [42,43]. Here tamoxifen treatment resulted in a significant (*P* < 0.05) loss in the body weight of mice fed the control diet, compared to untreated mice fed the control diet (Figure 2E). Mice fed the Fe-Lf diet and treated with tamoxifen had significantly (*P* < 0.05) higher body weights compared to mice fed the control diet and treated with tamoxifen.

### Oral Fe-Lf attenuates tamoxifen-induced immunosuppression as evidenced by restoration of IL-18 and IFN-γ expression

**Figure 3:**
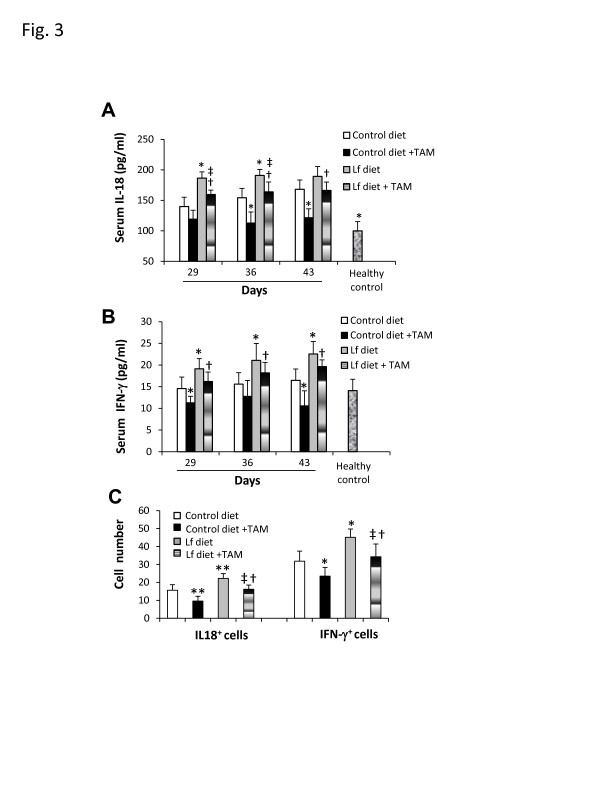
**Inhibition of reductions of IL-18 and IFN-γ in sera and intestinal cells.** Blood and intestinal samples were collected from the mice in the prevention when they were killed on days 29, 36 and 43**. A**,**B**: The serum levels of IL-18 (**A**) and IFN-γ (**B**) were measured in the above mice and a group of 6 healthy control mice. **C**: The intestinal tissues were sectioned, immunostained with Abs against mouse IL-18 and IFN-γ, respectively, and examined by microscopy. IL-18^+^ and IFN-γ^+^ cells were counted in 10 fields. Results are expressed as the mean value ± SD. “*” *P* < 0.05 and “**” *P* < 0.001 versus the group fed the control diet, “†” *P* < 0.05 versus the group fed the control diet and treated with tamoxifen, and “‡” *P* < 0.05 versus the group fed the Fe-Lf diet.

Blood samples were collected from the mice (n = 6, per group) sacrificed on days 29, 36 and 43 in the prevention experiment (Figure 2A). As shown in Figure 3A, mice bearing 4T1 tumors had significantly higher levels of serum IL-18 than the healthy control mice, at all the indicated time points. In contrast, the serum levels of IL-18 in mice fed the control diet and treated with tamoxifen were significantly (*P* < 0.05) lower on day 36 and 43 than those of untreated mice fed the control diet. Feeding of the Fe-Lf diet significantly (*P* < 0.05) elevated the serum IL-18 levels on days 29 and 36, compared to the control diet. The serum levels of IL-18 in mice treated with the combination of Fe-Lf diet and tamoxifen were significantly higher on day 29 (*P* < 0.01), 36 (*P* < 0.01) and 43 (*P* < 0.001) than those of mice treated with the combination of the control diet and tamoxifen. Serum levels of IFN-γ (Figure 3B) showed a similar pattern of change to IL-18, but the levels of serum IFN-γ in mice bearing 4T1 tumors were the similar to those in healthy controls, and did not significantly increase over time.

Sections of the intestines of mice killed on day 43 in the prevention experiment (Figure 2A) were stained with Abs against IL-18 and IFN-γ, and the numbers of cells in the intestinal lamina propria expressing IL-18 and IFN-γ were enumerated. As shown in Figure 3C, tamoxifen therapy significantly (*P* < 0.001) reduced the number of IL-18^+^ cells in the lamina propria, whereas in contrast the Fe-Lf diet significantly (*P* < 0.001) increased the number of IL-18^+^ cells, compared to that of mice fed the control diet. Further, the Fe-Lf diet attenuated the reduction in the number of IL-18^+^ cells caused by tamoxifen therapy, resulting in a significantly (*P* < 0.05) higher number of IL-18^+^ cells in mice treated with the combination of Fe-Lf and tamoxifen than that of mice treated with the combination of control diet and tamoxifen. Similarly, tamoxifen therapy significantly (*P* < 0.05) reduced the number of IFN-γ^+^ cells in the lamina propria (Figure 3C). In contrast, the Fe-Lf diet significantly (*P* < 0.05) increased the number of IFN-γ^+^ cells, and attenuated the reduction in the number of IFN-γ^+^ cells in the lamina propria caused by tamoxifen therapy.

### Identity of cells in the intestinal lamina propria that contain high levels of Lf

**Figure 4:**
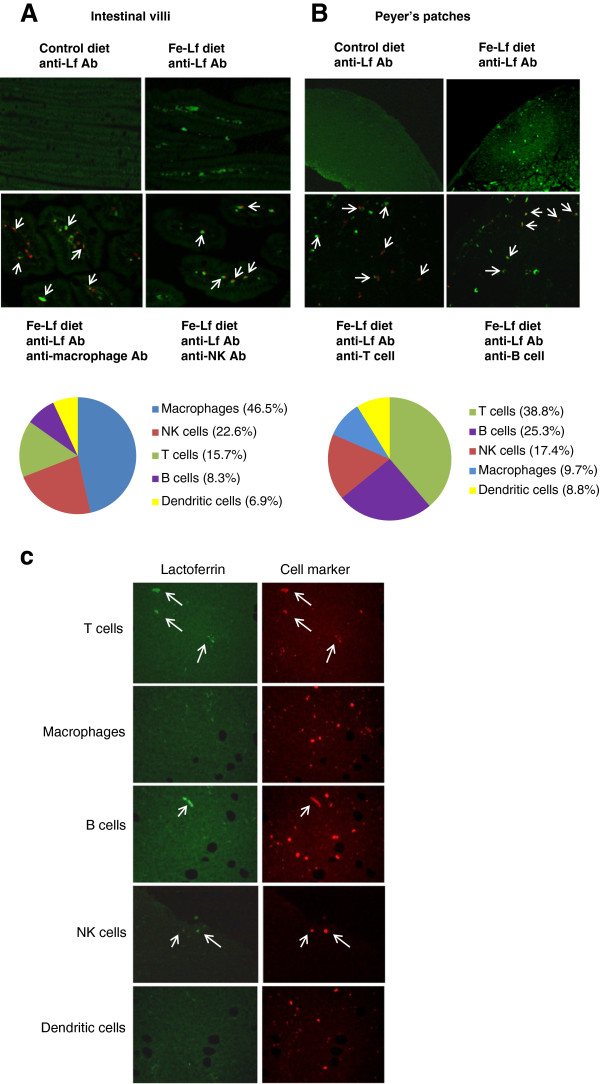
**Oral administration of Fe-Lf leads to high levels of Lf in leukocytes in the lamina propria and Peyer’s patches, which migrate to tumors. A**,**B**: Identification of leukocyte subsets in the lamina propria (**A**) and Peyer’s patches (**B**) that contain high levels of Lf. Representative illustrations were taken on day 43 of intestinal villus and Peyer’s patch sections, respectively, from mice in the prevention experiment fed with control and Fe-Lf diets. Sections were immunostained with FITC-conjugated (green) Abs against bovine Lf. The anti-Lf Ab-stained intestinal villus sections were further stained with Abs against markers for macrophages, NK cells, T cells, B cells and dendritic cells. Illustrated are (**A**) intestinal sections double-stained with an anti-Lf Ab (green), and Abs (red) against macrophages and NK cells, and (**B**) Peyer’s patch sections double-stained with an anti-Lf Ab (green), and Abs (red) against T cells and B cells. Magnification, x200. Arrows point to the double-stained cells. The number of double-stained cells of each leukocyte subset was counted, and the percentage of each subset was calculated and plotted as a pie chart. **C**: Phenotyping of Lf-laden leukocytes that infiltrate the tumors of mice fed the Fe-Lf diet. Sections of tumors on day 29 taken from mice in the prevention experiment fed the control or Fe-Lf diets. Tumor sections were immunostained with a FITC-conjugated (green) anti-Lf Ab (left panel), followed by Abs (red) against leukocyte subset markers (right panel) for T cells, macrophages, B cells, NK cells and dendritic cells, as indicated.

We previously demonstrated that bovine Fe-Lf is taken up by cells residing in the lamina propria and Peyer’s patches [36]. In agreement, cells of the intestinal villi of mice fed the Fe-Lf diet contained high levels of Lf (Figure 4A), as compared to mice fed the control diet. Intestinal villus sections were double-stained with an Ab against bovine Lf and Abs against different leukocyte markers, followed by a FITC or alexa fluor 568-conjugated secondary Ab, respectively, to identify the cells that contained high levels of Lf. Representative illustrations show macrophages, and NK cells that contain high levels of Lf. The percentages of each leukocyte subset that had high levels of Lf were calculated (Figure 4A), indicating that macrophages, NK and T cells within the intestinal lamina propria contained the highest levels of Lf.

### Identity of cells in Peyer’s patches that contain high levels of Lf

Peyer’s patches of mice fed the Fe-Lf diet contained high levels of Lf (Figure 4B), as compared to mice fed the control diet. Representative illustrations show CD3^+^ T cells, and B cells that contained high levels of Lf. The percentages of each leukocyte subset that had high levels of Lf were calculated (Figure 4B), indicating that B and T cells in the Peyer’s patches contained the highest levels of Lf.

### Intestinal leukocytes that contain high levels of Lf migrate to distal tumors

The possibility that intestinal leukocytes which contained high levels of Lf might migrate to distal tumors was examined. 4T1 breast tumors were collected from mice in the prevention experiment (Figure 2A) which had been fed for 28 days with the control diet or the Fe-Lf diet. Tumor sections were double-stained with Abs against various leukocyte subset markers, and against bLf. Very few leukocytes could be detected in the tumors of mice fed the control diet (data not shown). In contrast, leukocytes were readily detected in the tumors of mice fed the Fe-Lf diet (Figure 4C). T, B and NK cells present in tumors stained positively for bLf, suggesting that these three types of leukocytes, but not macrophages and dendritic cells, had migrated to the tumors from the intestine.

## Discussion

The present study has demonstrated that oral administration of Fe-Lf improves tamoxifen therapy in a mouse model of basal-like breast cancer, and overcomes tamoxifen-mediated immunosuppression. Orally fed Fe-Lf augmented tamoxifen therapy to delay the appearance of palpable tumors in the breasts of female Balb/c mice, and inhibited their subsequent growth. It augmented tamoxifen-mediated inhibition of the metastasis of tumors to the liver and lung. Oral Fe-Lf increased serum levels of IL-18 and IFN-γ and the numbers of cells expressing IL-18 and IFN-γ in intestinal tissues, and prevented their reduction by tamoxifen. It attenuated the loss of body weight caused by tamoxifen and cancer cachexia.

The 4T1 tumor cell line employed here was derived from the 410.4 cell line obtained from a spontaneously arising mouse mammary epithelial tumor [41,44], and hence cannot be regarded as a tumor that has been forced to acquire tamoxifen-resistance. It is resistant to the effects of tamoxifen by virtue of the fact that it intrinsically expresses very low levels of ER [37], and accordingly does not respond to estrogen [38]. Thus, the study describes the impact of co-treatment with Lf and tamoxifen on the development of established ER^-ve^ disease.

The anti-tumor activity of Lf is largely dependent on its ability to stimulate anti-tumor immunity when taken orally, by promoting both innate and adaptive immune responses [33,45]. Each of the Fe-Lf-induced cytokines IL-18 and IFN-γ might be expected to play a role in Fe-Lf-mediated antitumor immunity. Thus, orally administered Lf was previously reported to exhibit antitumor activity through production of IL-18 in the intestinal mucosa [46]. Iron-saturated bLf was chosen for the current study, as we have previously shown that it has superior antitumor activity compared to native bLf when combined with chemotherapeutic agents [36]. When fed to C57BL6 mice bearing a variety of different tumor types, it increased antitumor cytotoxicity, tumor apoptosis and the infiltration of tumors by leukocytes. It bound to the intestinal epithelium and was preferentially taken up within Peyer’s patches. It increased the production of Th1 and Th2 cytokines within the intestine and tumor, including TNF, IFN-γ, as well as nitric oxide that have been reported to sensitize tumors to chemotherapy. Importantly, it restored both red and white peripheral blood cell numbers depleted by chemotherapy, potentially fortifying the mice against cancer [36]. The presence of iron may have several beneficial effects, including rendering Fe-Lf more resistant to proteolysis as it passes through the gastrointestinal tract [47], and enhancing lymphocyte function [48].

Here we further demonstrated that oral ingestion of bovine Fe-Lf leads to increases in the Lf content of macrophages, NK and T cells in the intestinal lamina propria, and B and T cells in Peyer’s patches. An interesting phenomenon was the finding that many of the T and B cells, and NK cells that infiltrated into tumors in response to feeding of Fe-Lf contained high levels of Lf. The most plausible explanation is that these cells are derived from the populations of cells in the intestine that contain high levels of Lf [36,49].

Reanalysis of the results of the Royal Marsden Hospital study of primary breast cancer prevention [50] showed that obese women treated with tamoxifen gained significantly less body weight over a 6-year period than obese women given placebo, indicating that tamoxifen can cause weight loss [51]. Tamoxifen has an effect on energy homeostasis in rodents such that it markedly decreases food intake and body weight [42,43]. Tamoxifen-induced anorexia in rats was associated with fatty acid synthase inhibition in the ventromedial nucleus of the hypothalamus and accumulation of malonyl-CoA [51]. Tamoxifen induces rapid atrophy and metaplasia in mouse stomach [52]. In the present study, body weight loss was observed, particularly in the first week after tamoxifen administration in mice fed the control diet. The Fe-Lf diet attenuated the body weight loss induced by tamoxifen.

The 4T1 model of metastatic breast cancer represents a model of cancer cachexia, which is a serious problem for cancer patients as it physically weakens patients and reduces their response to treatment. Here, we also showed that oral Fe-Lf attenuated cancer cachexia, as evidenced by reduced loss of body weight, and increased weights of gastrocnemius muscle and ovarian adipose tissue in tumor-bearing animals. The anti-tumor activity of Fe-Lf may partly contribute to the ability to inhibit cachexia, as the sizes of primary and metastatic tumors were significantly smaller in Fe-Lf-treated mice, thus reducing energy wasting by tumor cells. Effects of Fe-Lf in preventing fatty acid synthase inhibition and accumulation of malonyl-CoA in the hypothalamus, and stomach atrophy may also contribute, but additional studies will be required to determine their relevance.

Tamoxifen forms DNA adducts in human colon after administration, and may elevate the risk of gastrointestinal cancers [53]. It inhibits the growth of normal human colon epithelial cells [54]. bLf has the potential to improve the overall physiological condition due to its beneficial effects on the gut epithelium, as it has been shown to inhibit chemically-induced carcinogenesis in the colon [55], and human and bovine Lf stimulate the proliferation and differentiation of crypt cells and enterocytes [56,57]. It improves the microbial intestinal environment by inhibiting the growth of pathogens and stimulating the establishment of beneficial microflora [58].

The dose of Fe-Lf used in the present study equates to a readily consumable dose of 2.5 g/day for humans, based on equivalent surface area. The dose of tamoxifen used equates to a human dose of 25 mg every two days, which is slightly less than the 20 mg/day dose given to breast cancer patients. The results indicated that Fe-Lf was slightly superior to tamoxifen in inhibiting the growth of tumors, albeit the difference was not significant. The added effects of tamoxifen and Fe-Lf in inhibiting the growth of ER^-ve^ 4T1 cells can be explained by the chemotherapeutic properties of tamoxifen, and the ability of Fe-Lf to stimulate anti-tumor immunity and overcome tamoxifen-induced immunosuppression.

## Conclusions

In conclusion, Fe-Lf augments the chemotherapeutic efficacy of tamoxifen in the treatment of ER^-ve^ breast cancer as evidenced by a delay in tumor formation, and inhibition of tumor growth and metastasis. It overcomes tamoxifen-induced impairment of the immune response, and attenuates body weight loss due to tamoxifen therapy and cancer-associated cachexia, thus fortifying the tumor-bearing mice. Iron-enriched bovine Lf is a comparatively inexpensive natural product, which has proven to exhibit no adverse toxicity [59]. The above panoply of features indicate that bovine Fe-Lf might be a promising and attractive supplement to add to tamoxifen treatment to delay or inhibit cancer relapse due to the outgrowth of tumors that no longer respond to the anti-estrogenic effects of tamoxifen.

## Abbreviations

ANOVA: Analysis of variance; bLf: Bovine lactoferrin; BSA: Bovine serum albumin; CTL: Cytotoxic T lymphocytes; DMSO: Dimethyl sulfoxide; ER: Estrogen receptor; ERK: Extracellular-signal-regulated kinase; Fe-Lf: Iron-saturated lactoferrin; FITC: Fluorescein isothiocyanate; HE: Haematoxylin and eosin; HER2: Human epidermal growth factor receptor 2; IFN: Interferon; IGF-1R: Insulin-like growth factor 1 receptor; IL: Interleukin; LAK: Lymphokine-activated killer; LDH: Lactate dehydrogenase; MTT: Methyl thiazolyl tetrazolium; NK: Natural killer; OD: Optical density; PBMC: Peripheral blood mononuclear cells; PI3K: Phosphatidylinositol 3-kinase; PR: Progesterone receptor; TGF: Transforming growth factor; TNF: Tumor necrosis factor; TRAIL: Tumor necrosis factor-related apoptosis-inducing ligand.

## Competing interests

X. Sun, K.P. Palmano, and G.W. Krissansen are inventors on a related patent application. No potential conflicts of interest are disclosed by the other authors.

## Authors’ contributions

XS, AP, RJ and SR performed the animal studies. XS contributed to the design of experiments, performed the animal studies and the experimental analysis of the results, contributed to data interpretation, and drafted the manuscript. KPP was involved in preparation of Fe-Lf, and contributed to data interpretation and manuscript writing. GWK conceived of the study, participated in its design and coordination, interpretation of data, and writing of final manuscript. All authors read and approved the final manuscript.

## Pre-publication history

The pre-publication history for this paper can be accessed here:

http://www.biomedcentral.com/1471-2407/12/591/prepub
